# Spectrophotometric and Multivariate Calibration Techniques for Simultaneous Determination of Different Drugs in Pharmaceutical Formulations and Human Urine: Evaluation of Greenness Profile

**DOI:** 10.1155/2020/8873003

**Published:** 2020-05-29

**Authors:** Ahmed Mostafa

**Affiliations:** Department of Pharmaceutical Chemistry, College of Clinical Pharmacy, Imam Abdulrahman Bin Faisal University, King Faisal Road, P.O. Box 1982, Dammam 31441, Saudi Arabia

## Abstract

Eco-friendly, rapid, and cost-effective two spectrophotometric methods were developed and validated for the determination of atenolol, paracetamol, hydrochlorothiazide, and levofloxacin. The first method is the newly developed extended derivative ratio (EDR) and the second method is multivariate curve resolution—alternating least squares (MCR-ALS). In the EDR method, the extended derivative ratio amplitudes at 281.6, 237.6, 279.2, and 282.8 nm were used for quantification of atenolol, paracetamol, hydrochlorothiazide, and levofloxacin, respectively. In the MCR-ALS method, calibration model was developed and correlation constraint was employed. External validation data set composed of seven mixtures was used, and different figures of merits such as root mean square error of prediction, standard error of prediction, bias, and relative error of prediction were calculated, and satisfactory results were obtained. Both methods provided comparable results. The methods were validated and applied for the determination of the target analytes in dosage forms, spiked and real human urine. Thereafter, the obtained results were statistically compared to the published methods and revealed no significant difference regarding accuracy and precision. Furthermore, the greenness profile of the methods was evaluated using the National Environmental Methods Index “NEMI” and Analytical Eco-Scale. The developed methods can be used as a valid eco-friendly and simple cost-effective alternative to the commonly used chromatographic methods for the routine analysis of the studied drugs in dosage forms and human urine.

## 1. Introduction

**Figure 1:**
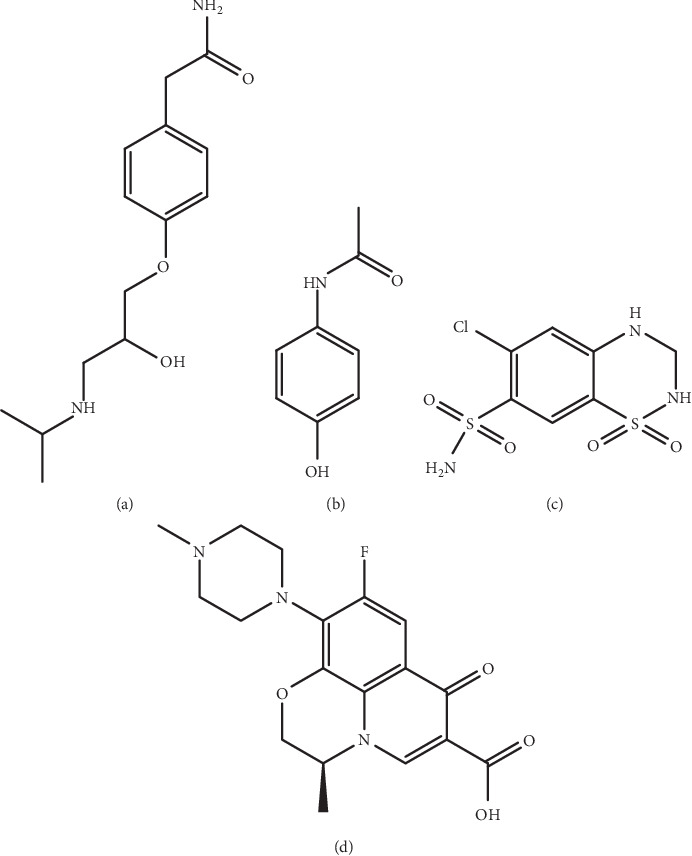
Chemical structures of atenolol (a), paracetamol (b), hydrochlorothiazide (c), and levofloxacin (d).

Atenolol (AT) (Figure 1(a)) is a selective ß1 receptor blocker and is used for the treatment of hypertension and coronary heart disease. AT undergoes little or no metabolism in the liver and is mainly excreted in urine (over 85% of the absorbed drug is excreted in urine as unchanged drug) [1]. Different analytical techniques have been reported for the analysis of AT such as high performance liquid chromatography (HPLC) [2–4], spectrophotometric [2, 5, 6], capillary electrophoresis [7, 8], gas chromatography (GC) [9], and spectrofluorimetric [10] methods.

Paracetamol (PR) or acetaminophen (Figure 1(b)) is an over-the-counter (OTC) analgesic and antipyretic drug. PR is metabolized in liver and mainly excreted in urine. Around 1–4% of the administered dose is excreted unchanged [11]. However, 47–62% of the dose is excreted as paracetamol conjugated to glucuronide and 25–36% as sulphate conjugates ([12, 13]. A small amount (8–10%) is metabolized through oxidation to form cysteine and mercapturic acid conjugates [13].

Various studies can be found in the literature for the analysis of PR including HPLC [14–17], GC [18], spectrophotometry [19, 20], voltammetry [21], and electrophoresis [22].

Hydrochlorothiazide (HZ) (Figure 1(c)) is a thiazide diuretic that is used to treat hypertension and edema associated with certain disease conditions such as congestive heart failure. HZ is excreted (>95%) as unchanged drug in urine [23, 24]. Different techniques have been reported for HZ quantitation such as HPLC [25–28], GC [29], spectrophotometry [30, 31], and voltammetry [32].

Levofloxacin (LV) (Figure 1(d)) is a fluoroquinolone antibiotic that inhibits DND topoisomerase IV and gyrase in Gram-positive and Gram-negative bacteria, respectively. It is used for urinary, respiratory, skin, and soft tissue infections [33]. Several reports are found in the literature for the determination of LV including different techniques such as HPLC [34, 35], spectrophotometry [34, 36], voltammetry [37], and capillary electrophoresis [38].

AT, PR, HZ, and LV can be coadministered together by some patients, and they are excreted mainly in the urine. To the best of our knowledge, no method has been reported for the simultaneous determination of the four drugs.

Green analytical chemistry (GAC), which is concerned with developing new methods that are sustainable and more environmentally friendly, has gained much interest among analytical chemists [39, 40]. However, the challenge is to achieve a compromise between increasing the quality of the developed methods and improving the method greenness [39, 41]. UV-Vis spectroscopy is considered a greener analytical technique when compared to HPLC. UV-Vis spectroscopy is a fast technique that consumes low solvent volumes, thus reducing the amount of generated waste. In addition, this technique is cost-effective and does not need expensive solvents or sophisticated instruments [42, 43]. However, the major challenge when using UV-Vis spectroscopy is the analysis of multicomponent mixtures, especially when the analytes spectra are strongly overlapped [42, 44]. To overcome this challenge, the author proposed the use of nonconventional spectrophotometric technique, namely, extended derivative ratio (EDR). In addition, the use of multivariate calibration helped in the mathematical resolution of the multicomponent mixture.

Therefore, this work aimed to develop green, cost-effective, and precise methods for the analysis of AT, PR, HZ, and LV. Two methods including a nonconventional univariate method (i.e., extended derivative ratio (EDR)) and a multivariate method (i.e., multivariate curve resolution—alternating least squares (MCR-ALS)) were developed and validated and then successfully applied for analyzing the four analytes in different pharmaceutical dosage forms, spiked and real human urine. In addition, the method greenness was assessed using the National Environmental Methods Index (NEMI) [45, 46] and the analytical Eco-Scale [47].

### 1.1. Theoretical Background

#### 1.1.1. Extended Derivative Ratio Technique (EDR)

Let's consider mixture *M* composed of four compounds (*W*, *X*, *Y,* and *Z*). Once Beer's law is obeyed over the whole wavelength range, the absorption spectrum of *M* will be equal to the sum of the individual spectra of the four analytes (i.e., the additive property of Beer's law). It can be defined by the following equation:

(1) $$\begin{array}{c} A_{M\;\lambda_{i}}=\varepsilon_{W\;\lambda_{i}}C_{W}+\varepsilon_{X\;\lambda_{i}}C_{X}+\varepsilon_{Y\;\lambda_{i}}C_{Y}+\varepsilon_{Z\;\lambda_{i}}C_{Z}, \end{array}$$

where *A*_*Mλ*_*i*__ is the absorbance of *M* at wavelength *λ*_*i*_, *ε*_*Wλ*_*i*__, *ε*_*Xλ*_*i*__, *ε*_*Yλ*_*i*__ and *ε*_*Zλ*_*i*__ are the molar absorptivity of compounds *W*, *X*, *Y,* and *Z* at wavelength  *λ*_*i*_ and *C*_*W*_, *C*_*X*_, *C*_*Y*_, and *C*_*Z*_ are the concentrations of *W*, *X*, *Y,* and *Z*, respectively.

For compound *W* determination, a mixture (*m*_1_) containing all other three compounds (*X*, *Y,* and *Z*) except compound *W* is scanned and it can be represented by the following equation:

(2) $$\begin{array}{c} A^{\prime }{}_{m1\lambda_{i}}=\varepsilon_{X\lambda_{i}}C{{}^{\circ}}_{X}+\varepsilon_{Y\lambda_{i}}C{{}^{\circ}}_{Y}+\varepsilon_{Z\lambda_{i}}C{{}^{\circ}}_{Z}. \end{array}$$

For both equations (1) and (2) the optical path is considered to be 1 cm.

Dividing the mixture spectrum *M* by *m*_1_ will result in the following ratio spectrum:

(3) $$\begin{array}{c} \frac{A_{M\;\lambda_{i}}}{A^{\prime }{}_{m1\;\lambda_{i}}}=\frac{\varepsilon_{W\lambda_{i}}C_{W}+\varepsilon_{X\lambda_{i}}C_{X}+\varepsilon_{Y\lambda_{i}}C_{Y}+\varepsilon_{Z\lambda_{i}}C_{Z}}{\varepsilon_{X\lambda_{i}}C{{}^{\circ}}_{X}+\varepsilon_{Y\lambda_{i}}C{{}^{\circ}}_{Y}+\varepsilon_{Z\lambda_{i}}C{{}^{\circ}}_{Z}}\\ =\frac{\varepsilon_{W\lambda_{i}}C_{W}}{\varepsilon_{X\lambda_{i}}C{{}^{\circ}}_{X}+\varepsilon_{Y\lambda_{i}}C{{}^{\circ}}_{Y}+\varepsilon_{Z\lambda_{i}}C{{}^{\circ}}_{Z}}+\frac{\varepsilon_{X\lambda_{i}}C_{X}+\varepsilon_{Y\lambda_{i}}C_{Y}+\varepsilon_{Z\lambda_{i}}C_{Z}}{\varepsilon_{X\lambda_{i}}C{{}^{\circ}}_{X}+\varepsilon_{Y\lambda_{i}}C{{}^{\circ}}_{Y}+\varepsilon_{Z\lambda_{i}}C{{}^{\circ}}_{Z}}\\ =\frac{\varepsilon_{W\;\lambda_{i}}C_{W}}{\varepsilon_{X\lambda_{i}}C{{}^{\circ}}_{X}+\varepsilon_{Y\lambda_{i}}C{{}^{\circ}}_{Y}+\varepsilon_{Z\lambda_{i}}C{{}^{\circ}}_{Z}}+\text{K}, \end{array}$$

where K is constant.

The derivative ratio spectra can be represented as follows:

(4) $$\begin{array}{c} \frac{d}{d\lambda }\left[\frac{A_{M{\;\lambda }_{i}}}{A{`}_{m1{\;\lambda }_{i}}}\right]=\frac{d}{d\lambda }\left[\frac{\varepsilon_{W{\;\lambda }_{i}}C_{W}}{\varepsilon_{X{\;\lambda }_{i}}C{{}^{\circ}}_{X}+\varepsilon_{Y{\;\lambda }_{i}}C{{}^{\circ}}_{Y}+\varepsilon_{Z{\;\lambda }_{i}}C{{}^{\circ}}_{Z}}\right]=\frac{d}{d\lambda }\left[\frac{\varepsilon_{W{\;\lambda }_{i}}}{\varepsilon_{X{\;\lambda }_{i}}C{{}^{\circ}}_{X}+\varepsilon_{Y{\;\lambda }_{i}}C{{}^{\circ}}_{Y}+\varepsilon_{Z{\;\lambda }_{i}}C{{}^{\circ}}_{Z}}\right]C_{W} \end{array}$$

Equation (4) indicates that the derivative ratio spectrum amplitude is dependent and directly proportional to the concentration of the compound *W*. Therefore a calibration curve can be drawn by dividing the spectra of various concentrations of a pure compound *W* by the ternary mixture of the other three compounds, and a regression equation can be obtained. Similarly, the other three compounds can be determined using the same procedure as for compound *W*.

#### 1.1.2. Multivariate Curve Resolution—Alternating Least Squares (MCR-ALS)

MCR is a soft-modeling algorithm that can extract relevant information of the pure components in multicomponent systems. It can be performed through the bilinear decomposition of the data matrix *D* as follows:

(5) $$\begin{array}{c} D=CS^{T}+E, \end{array}$$

where *C* and *S*^*T*^ are the pure concentration and spectral profiles [48]. *E* is the residuals matrix of the data not explained by the bilinear model. The *C* and *S*^*T*^ profiles can be optimized iteratively using Alternating Least Squares (ALS) until a certain convergence criterion is achieved. The initial estimates of the components' spectra were used to start the optimization process, and simple–to-use interactive self-modeling mixture analysis (SIMPLISMA) [49] was employed. Specific constraints such as nonnegativity, closure, unimodality, and correlation constraints [50, 51] can be applied during the optimization process. In the presented work, nonnegativity spectra constraint, nonnegativity concentration constraint, and correlation constraint were applied. The latter constraint enhances building a calibration model that allows the quantitative analysis of components in the presence of unknown interferences [50, 52].

The MCR-ALS model's consistency and reliability can be measured using the percentage of lack of fit (Equation (6)) and the percentage of total explained variance (Equation (7)). The following equations help to calculate the two parameters:

(6) $$\begin{array}{c} \text{lack of fit }\left(\%\right)=100\sqrt{\frac{\sum_{i.j}e_{ij}^{2}}{\sum_{i.j}d_{ij}^{2}}}, \end{array}$$

(7) $$\begin{array}{c} S^{2}\;=100\sqrt{\frac{\sum_{i.j}d_{ij}^{2}\;-\;\sum_{i.j}e_{ij}^{2}}{\sum_{i.j}d_{ij}^{2}}}, \end{array}$$

where *d*_*ij*_ is an element of the data matrix *D* and *e*_*ij*_ is the associated residual (the difference between experimental data input and model reproduced data).

*(1) Validation of the Model*. The performance of the developed model will be evaluated, employing a data set composed of 7 mixtures used as an external validation set. Different figures of merit were calculated to evaluate the obtained results according to the following equations [50]:

Root mean square error of prediction (RMSEP),

(8) $$\begin{array}{c} \text{RMSEP}=\;\sqrt{\frac{\sum_{i=1}^{n}{\left(c_{i}-{\hat{c}}_{i}\right)}^{2}}{n}}, \end{array}$$

Bias,

(9) $$\begin{array}{c} \text{bias}=\;\frac{\sum_{i=1}^{n}\left(c_{i}-{\hat{c}}_{i}\right)}{n}. \end{array}$$

Standard error of prediction (SEP),

(10) $$\begin{array}{c} \text{SEP}=\;\sqrt{\frac{\sum_{i=1}^{n}{\left(c_{i}-{\hat{c}}_{i}-\text{bias}\right)}^{2}}{n-1}}. \end{array}$$

Relative percentage error in the concentration predictions (RE %),

(11) $$\begin{array}{c} \text{RE }\left(\%\right)=100\sqrt{\frac{\sum_{i=1}^{n}{\left(c_{i}-{\hat{c}}_{i}\right)}^{2}}{\sum_{i=1}^{n}c_{i}^{2}}}, \end{array}$$

where *c*_*i*_ and ${\hat{c}}_{i}$ are the known and predicted analyte concentration in sample *i*, respectively, and *n* is the total number of validation samples.

Furthermore, a linear regression fit was performed between the known and predicted concentrations, and slope, intercept, and determination coefficients were calculated.

## 2. Material and Methods

### 2.1. Instrumentation and Software

Shimadzu UV–1800 double-beam spectrophotometer (Tokyo, Japan) with 1 cm quartz cells was used for data acquisition. Scans were recorded in the wavelength range of 200–330 nm at 0.2 nm intervals. Spectra were acquired by Shimadzu UV-Probe version 2.62. The MCR model was developed using MCR-ALS GUI 2.0 software for use with Matlab 2015a [53]. The software is freely available at http://www.mcrals.info.

### 2.2. Chemicals and Reagents

Atenolol (CAS no. 29122-68-7; 98% minimum purity), paracetamol (CAS no. 103-90-2; 98% minimum purity), and hydrochlorothiazide (CAS no. 58-93-5; 98% minimum purity) were purchased from Sigma–Aldrich (Steinheim, Germany). Levofloxacin hemihydrate (CAS no. 138199-71-0; 99% minimum purity) was purchased from Santa Cruz Biotechnology Inc. (Santa Cruz, Canada). *Helix pomatia β*-glucuronidase enzyme used for hydrolysis was obtained from Sigma–Aldrich (St. Louis, USA).

Ethanol (LiChrosolv®, HPLC grade) was obtained from Merck (Darmstadt, Germany). Ultrapure water (18.2 MΩ) was obtained by Pure Lab Ultra water system (ELGA, High Wycombe, United Kingdom) and used for all sample preparation.

This study was approved by the Research and Ethics Committee of the College of Clinical Pharmacy, Imam Abdulrahman Bin Faisal University, Saudi Arabia. Upon receiving informed written consent, blank urine samples were collected from three healthy volunteers (23–25 years old) and were kept at −20°C.

Commercial pharmaceutical dosage forms of the studied drugs were purchased from the local market and they were as follows:

- Tenormin® tablets (batch # PF742) produced by AstraZeneca UK Limited, United Kingdom and labeled to contain 100 mg AT per tablet.
- Panadrex® tablets (batch # JT377) produced by Kuwait Saudi Pharmaceutical Industries Co, Kuwait and labeled to contain 500 mg PR per tablet.
- HCT Georetic 25® tablets (batch # 1832207) produced by Marcyrl Pharmaceutical Industries, Egypt and labeled to contain 25 mg HZ per tablet.
- Tavanic® tablets (batch # 7PK7A) produced by Sanofi Winthrop Industries, France and labeled to contain 500 mg LV per tablet.

### 2.3. Procedures

#### 2.3.1. Standard Solutions

Stock solutions were prepared by dissolving 10 mg of AT, PR, HZ, and LV separately in 10 mL ethanol and were used as stock standard solutions for the EDR and MCR-ALS methods. All solutions were stored at 4°C and were stable for at least three months. Working solutions were obtained by appropriate dilutions using ultrapure water to reach the calibration range of each method.

#### 2.3.2. Spectroscopic Characteristics

The UV spectra of solutions comprising 10 *μ*g mL^−1^ of AT and 5 *μ*g mL^−1^ of PR, HZ and LV were analyzed separately over the wavelength range of 200–330 nm.

#### 2.3.3. EDR Method

Appropriate volumes of each stock standard solution of AT, PR, HZ and LV were transferred into four different sets of 10-mL volumetric flasks and diluted with ultrapure water to reach the calibration range of 5–40, 1–25, 1–15, and 1–15 *μ*g mL^−1^ for AT, PR, HZ, and LV, respectively. The absorption spectra were measured over the range of 200 to 330 nm using ultrapure water as blank.

*(1) Determination of AT*. A mixture composed of 7, 2, and 4 *μ*g mL^−1^ of PR, HZ, and LV, respectively, was scanned against ultrapure water as blank and spectra were stored (AT divisor). The UV spectra of working standard solutions of AT were divided by the stored divisor, and the second derivative of the resulting ratio spectra was calculated employing Δ*λ* = 4 nm and 10 as a scaling factor. The amplitudes at 281.6 nm were directly proportional to AT concentration. The calibration curve was obtained and the corresponding regression equation was calculated.

*(2) Determination of PR*. A mixture composed of 15, 3, and 5 *μ*g mL^−1^ of AT, HZ, and LV, respectively, was scanned against ultrapure water as blank and spectra were stored (PR divisor). The UV spectra of working standard solutions of PR were divided by the stored divisor, and the first derivative of the resulting ratio spectra was calculated employing Δ*λ* = 4 nm and 10 as a scaling factor. The amplitudes at 237.6 nm were directly proportional to PR concentration. The calibration curve was obtained, and the corresponding regression equation was calculated.

*(3) Determination of HZ*. A mixture composed of 15, 10, and 5 *μ*g mL^−1^ of AT, PR, and LV, respectively, was scanned against ultrapure water as blank and spectra were stored (HZ divisor). The UV spectra of working standard solutions of HZ were divided by the stored divisor, and the first derivative of the resulting ratio spectra was calculated using Δ*λ* = 2 nm and 10 as a scaling factor. The amplitudes at 279.2 nm were directly proportional to HZ concentration. The calibration curve was obtained, and the corresponding regression equation was calculated.

*(4) Determination of LV*. A mixture composed of 15, 7, and 2 *μ*g mL^−1^ of AT, PR, and HZ, respectively, was scanned against ultrapure water as blank and spectra were stored (LV divisor). The UV spectra of working standard solutions of LV were divided by the stored divisor, and the second derivative of the resulting ratio spectra was calculated employing Δ*λ* = 4 nm and 10 as a scaling factor. The amplitudes at 282.8 nm were directly proportional to the concentration of LV. The calibration curve was obtained, and the corresponding regression equation was calculated.

#### 2.3.4. MCR-ALS Method

Calibration and validation sets composed of 25 samples were constructed using a five-level four-factors design [54], in which five different concentration levels of AT, PR, HZ, and LV were introduced. The levels ranges were 5–25, 1–10, 1–10, and 1–7 *μ*g mL^−1^ for AT, PR, HZ, and LV, respectively. The UV spectra of all samples were scanned from 220 to 330 nm at 0.2 nm intervals. Eighteen samples were used to build the calibration model, while seven samples, randomly selected, were used for external validation. The absorption spectra were exported into Matlab to build the MCR-ALS calibration model using MCR-ALS GUI 2.0 software [53]. Fast nonnegativity least squares (for spectral and concentration profiles) and correlation constraints were employed.

### 2.4. Sample Preparation

#### 2.4.1. Analysis of Pharmaceutical Preparations

Ten tablets of each commercial formulation were weighed and pulverized. A portion equivalent to 100.0 mg of AT, PR, HZ, and LV were accurately added to a 100 mL conical flask and sonicated with 50 mL ethanol for 30 min. The solution was filtered into a 100 mL volumetric flask and completed to mark with ethanol. The procedures for EDR and MCR-ALS methods were followed as detailed under Sections 2.3.3 and 2.3.4, respectively.

#### 2.4.2. Analysis of Spiked and Real Human Urine Samples

All urine samples were syringe filtered through 0.45 *μ*m nylon filters. Aliquots of 100 *μ*L of blank urine were transferred into a set of 10 mL volumetric flasks. Different volumes of the stock standard solutions of AT, PR, HZ, and LV were added, vortex mixed, and the volume was completed to mark with ultrapure water. The procedures for EDR and MCR-ALS methods were followed as detailed under Sections 2.3.3 and 2.3.4, respectively. Analytes concentrations were calculated using the corresponding regression equations.

For method application in the analysis of real urine samples: (A) urine was collected 0–12 h from a healthy volunteer after the administration of a single oral dose of 500 mg PR, and (B) urine was collected 0–12 h from a healthy volunteer after the administration of 500 mg of LV. The collected urine volumes were accurately measured. A 100 *μ*L aliquot was diluted into 10 mL using ultrapure water and used for the direct determination of PR and LV.

## 3. Results and Discussion

**Figure 2:**
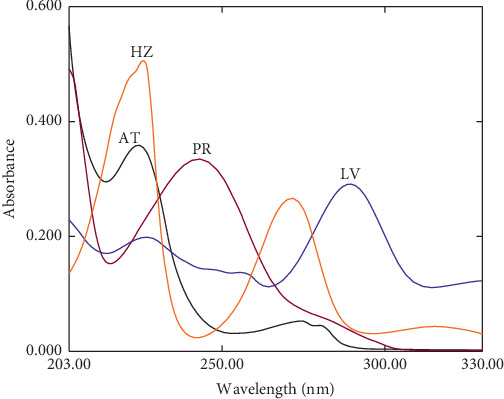
Zero-order absorption spectra of 10 *μ*g ml^−1^ atenolol (AT), 5 *μ*g ml^−1^ paracetamol (PR), 10 *μ*g ml^−1^ hydrochlorothiazide (HZ), and 5 *μ*g ml^−1^ levofloxacin (LV).

Figure 2 shows the zero-order absorption spectra of AT, PR, HZ, and LV. There is a severe overlap between the absorption bands of the four analytes over the whole wavelength range (200–330 nm). Thus, the simultaneous determination of these drugs is hindered using conventional calibration procedures without prior separation. Therefore, simple, reliable, and precise nonconventional univariate and multivariate chemometrics-assisted spectrophotometric methods were proposed for the simultaneous analysis of AT, PR, HZ, and LV in pharmaceutical dosage forms, spiked and real human urine samples. Furthermore, the developed methods' performance was evaluated and statistically compared with published methods [3, 16, 28, 34].

### 3.1. EDR Method

#### 3.1.1. Selection of Divisors and Derivative Parameters

The severe spectral overlap of the analytes of interest demonstrates the resolving power of the proposed method. Each analyte can be determined by using a divisor mixture composed of the other three analytes.

Six synthetic mixtures were prepared containing different concentration ratios of AT, PR, HZ, and LV within their linear ranges. The zero-order absorption spectra of these solutions were recorded and stored.

**Figure 3:**
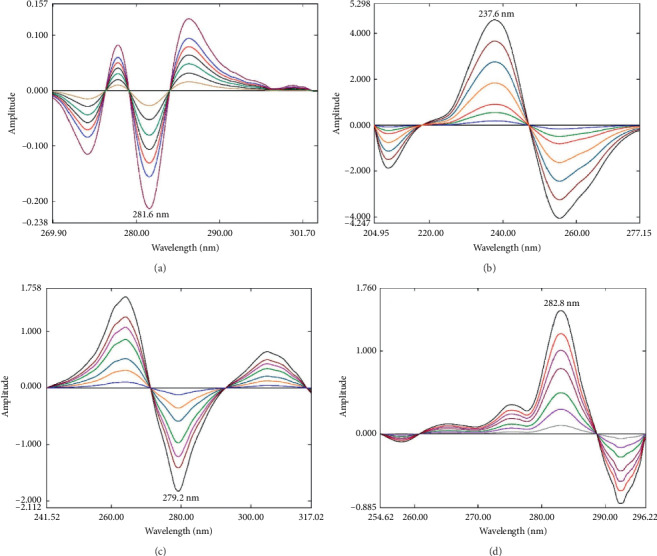
Overlay of the derivative ratio spectra obtained: (a) ^2^DD of AT (5–40 *μ*g mL^−1^); (b) ^1^DD of PR (1–25 *μ*g mL^−1^); (c) ^1^DD of HZ (1–15 *μ*g mL^−1^); and (d) ^2^DD of LV (1–15 *μ*g mL^−1^).

For determination of AT, the stored spectra of standard solutions containing AT, PR, HZ, and LV were divided by the stored AT divisor; then the second derivative of the obtained ratio spectra (^2^DD) was calculated using Δ*λ* = 4 nm and scaling factor 10 (Figure 3(a)). For the determination of AT, a reproducible amplitude was selected from the obtained derivative spectra. The calculated amplitude at 281.6 nm was found to be proportional to the concentration of AT.

On the other hand, for PR determination, the same procedures were followed and the ratio spectra were obtained as described above using PR divisor. Then, the first derivative of the ratio spectra (^1^DD) was calculated employing Δ*λ* = 4 nm and 10 as a scaling factor (Figure 3(b)). The calculated amplitude at 237.6 nm was found to be proportional to the concentration of PR.

Moreover, HZ was determined using similar procedures using the HZ divisor, and the ^1^DD was calculated employing Δ*λ* = 2 nm and 10 as a scaling factor (Figure 3(c)). The amplitude at 279.2 nm was measured and found to be proportional to the concentration of HZ.

Furthermore, LV was determined by using LV divisor and the ^2^DD was calculated using Δ*λ* = 4 nm and scaling factor of 10 (Figure 3(d)). The amplitude at 282.8 nm was calculated and found to be proportional to LV concentration.

The main parameters affecting the developed method performance were optimized for reliable and accurate determination of the analytes of interest. This included optimization of the divisor selection, which is a vital step in the proposed technique. The concentration of each analyte in the divisor was selected in a way that absorbances are low but meanwhile spectral features are evident [55]. Different mixtures were prepared and tested as divisor and the optimum one was selected. First and second derivatives of the ratio spectra were calculated and tested. The first derivative provided reliable and accurate results in case of PR and HZ. However, ^2^DD was preferred than ^1^DD in case of AT and LV because it provided better spectral resolution and more accurate and precise results. Moreover, the effect of the Δ*λ* for ^1^DD and ^2^DD was optimized and the best results were obtained using Δ*λ* = 2 for HZ and Δ*λ* = 4 for AT, PR, and LV. Furthermore, the scaling factor was optimized and a factor of 10 was found suitable for all analytes.

#### 3.1.2. Method Validation

Method validation was performed according to the International Conference on Harmonization (ICH) recommendations [56].

*(1) Linearity*. Seven concentrations were used in the concentration range of 5–40, 1–25, 1–15, and 1–15 *μ*g mL^−1^ of AT, PR, HZ, and LV, respectively. The developed method showed a high correlation coefficient (*r* ≥ 0.9994) and intercept value that was not statistically (*p* < 0.05) different from zero. Regression parameters are shown in Table 1.

*(2) Detection and Quantitation Limits*. Limit of detection (LOD) was calculated according to ICH as 3.3 (*σ*/*S*) and limit of quantification was calculated as 10(*σ*/*S*), where “*σ*” is the standard deviation of the intercept and “*S*” is the slope of the calibration curve (Table 1).

*(3) Accuracy and Precision*. Method accuracy was evaluated using three different concentrations of each drug analyzed in triplicate and percentage recovery of each analyte was calculated. Precision was evaluated by analyzing three different concentrations of each drug in triplicate within the same day (Intraday) and for three consecutive days (Interday) and relative standard deviation (%RSD) was calculated. The samples were analyzed according to the procedures under “2.3.3”. The results summed in Table 1 shows excellent recoveries and % RSD lower than 1.26 indicating the good accuracy and precision of the proposed method.

*(4) Selectivity*. Method selectivity was investigated using several laboratory synthetic mixtures comprising the four analytes in different concentration ratios within the linearity range. The results shown in Table 2 indicate the high selectivity of the EDR method.

*(5) Statistical Analysis*. The obtained results of the developed methods were compared with the published methods for the determination of AT [3], PR [16], HZ [28], and LV [34]. The results obtained in Table 3 revealed no significant difference between the proposed and the published methods with respect to accuracy and precision.

### 3.2. Multivariate Curve Resolution—Alternating Least Squares

The severe spectral overlap between the four analytes and the presence of unknown sample interferences in the urine matrix necessitate the use of multivariate calibration models to resolve such kind of mixtures. First-order multivariate calibration methods may be a good choice in such case only if the interfering background is well represented in the calibration phase. However, if such interferences are not represented in the calibration, first-order methods may not be a good choice. In such instances, second-order multivariate calibration models may work better due to their good prediction ability even in the presence of unknown interferences [57]. In this work, MCR-ALS model was developed for the determination of the four analytes of interest in pharmaceutical dosage forms and spiked human urine samples without including urine in the calibration samples.

#### 3.2.1. Calibration Set

Multilevel multifactor experimental design was employed to construct 25 mixtures of the four analytes thus providing mutual orthogonal factors [54]. Five different concentration levels, within the calibration range, were used for each analyte. A total of 18 mixtures were used to build the calibration model and 7 mixtures were utilized as an external validation set as shown in Table 4.

#### 3.2.2. Selection of the Wavelength Range

The quality of multivariate calibration relies on the wavelength range selected to build up the calibration model. Therefore, the data in the region 200–220 nm were discarded due to noise. Moreover, the band of 290–330 nm was excluded as well because AT had nonsignificant absorption in this region. Thus, the region of 220–290 nm with 0.4 nm interval was used in the developed MCR-ALS method.

#### 3.2.3. Developing the MCR-ALS Model

**Figure 4:**
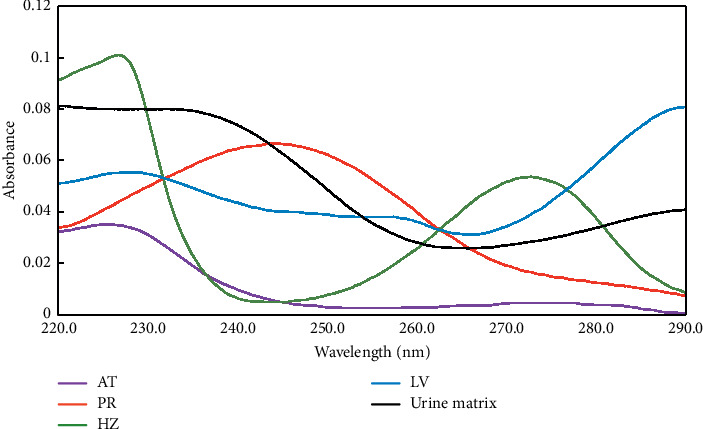
MCR-ALS resolved spectral profiles of the four target analytes (AT, PR, HZ, and LV) and the interfering urine matrix.

**Figure 5:**
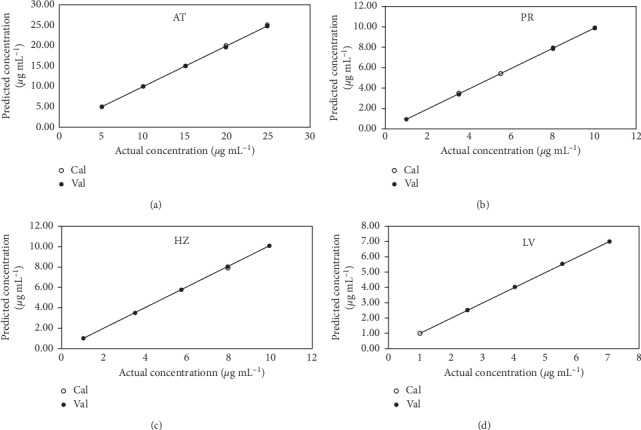
Scatter plot of actual drugs concentrations versus MCR-ALS predicted values of AT, PR, HZ, and LV.

No data preprocessing was conducted on the calibration matrix while developing the MCR-ALS model. To obtain a reasonable resolution by MCR-ALS model, initial estimation of the pure spectra of the target analytes was conducted. Five components were found to be responsible for the variations in different samples. Nonnegativity constraint (for spectral and concentration profiles) and correlation constraints were used in developing the model. The model resolved five species. The first four curves were very similar to AT, PR, HZ, and LV spectra with a correlation coefficient (*r*^2^  = 0.9996, 0.9998, 0.9997, and 09999, respectively) between the real and calculated AT, PR, HZ, and LV spectra. The fifth spectrum was estimated as the interfering urine matrix. The estimated spectral profiles are shown in Figure 4. The resolved matrix for each analyte was used to find the concentration profiles of each analyte and satisfactory results were obtained with a low lack of fit (% lof) of 0.1721. The developed model captured 99.99% of variance in the analyzed spectra.  Figure 5 shows the scatter plot of concentration values resolved by the MCR-ALS model versus the true concentration values. Good predictive ability of the model was obtained with determination coefficients of not less than 0.9998 for the four analytes.  Table 5 shows the different figures of merit of the developed model.

#### 3.2.4. Validation of the MCR-ALS Model

The developed model was applied on a series of external validation data set composed of 7 different synthetic mixtures within the calibration range of the analytes of interest (Table 4). The percent recoveries summarized in Table 6 show satisfactory results. To further validate the model, different figures of merit including RMSEP, SEP, RE%, and *r*^2^ were calculated for the external validation set. Satisfactory results were obtained as shown in Table 6. Moreover, accuracy, intraday and interday precision were calculated for the external validation set to further evaluate the model. Satisfactory results were obtained in terms of accuracy and precision (Table 6).

### 3.3. Analysis of Real Samples

#### 3.3.1. Analysis of Pharmaceutical Formulations

The developed methods were successfully used for the quantification of AT, PR, HZ, and LV in different pharmaceutical formulations and the results were statistically compared with the reported methods using paired *t*-test and F ratio at 95% confidence level. Satisfactory results were obtained showing no significant difference with the reported methods in terms of accuracy and precision (Table 7).

#### 3.3.2. Analysis of Spiked and Real Human Urine Samples

EDR and MCR-ALS methods were successfully employed for the quantification of the four analytes of interest in spiked human urine samples and satisfactory results were obtained (Table 8).

Pharmacokinetic studies on PR reported that it is excreted in urine mainly as conjugates [12, 13]. Therefore, PR urine sample was exposed to enzymatic hydrolysis to obtain the total PR (free + conjugated). An aliquot equivalent to 400 units of *β*-glucuronidase enzyme with sulfatase activity was added to 1 mL of the collected urine sample after oral administration of the PR dose. The pH was adjusted to 5 using acetate buffer and incubated at 37°C for 1 h [58]. The developed methods were applied to determine PR concentration for the same sample with and without enzymatic hydrolysis, before and after incubation. No significant difference that could be attributed to the increase in PR concentration due to enzymatic hydrolysis was obtained. This result provided evidence that the developed methods can determine total PR directly in urine without prior hydrolysis step. Furthermore, the published HPLC method [16] was employed to determine the total PR in the urine sample after enzymatic hydrolysis and the concentration determined was very closed from the concentration obtained using the developed methods with no enzymatic hydrolysis (Table 9).

Total PR excreted in urine was found in a concentration of 454.5 and 459.7 *μ*g mL^−1^ and a cumulative (0–12 h) urine excretion of 89.1 and 90.1% of the administered dose using EDR and MCR-ALS, respectively. This is in agreement with previous studies that reported similar PR urine excretion percentage [16, 20].

On the other hand, when urine sample B (LV dose) was analyzed using the developed methods, LV was determined with cumulative (0–12 h) urinary excretion of 60.5 and 62.4% of the administered dose using EDR and MCR-ALS, respectively. The results are in agreement with previous studies which reported similar LV excretion percentage [34]. Furthermore, the obtained results were compared to a published HPLC method [34] and showed no significant difference as shown in Table 9. The urinary excretion results of PR and LV are summarized in Table 9.

### 3.4. Assessment of the Methods Greenness

Greenness assessment of the developed methods was accomplished using two different methods, namely, NEMI and Analytical Eco-Scale.

#### 3.4.1. National Environmental Methods Index (NEMI)

This assessment method utilizes a greenness profile symbol composed of four quadrants representing method aspects according to the following criteria: any of the chemicals used is persistent, bioaccumulative or toxic (PBT), hazardous, corrosive (pH <2 or >12), and the amount of waste generated (>50 g). If the method is green, all four quadrants will be colored green. However, if the procedure does not meet any of these aspects, the corresponding quadrant will be left blank (i.e., uncolored).

In the proposed methods, ethanol, which is considered a green solvent, was used to prepare the stock solutions and water was used as a solvent for all further dilutions and spectrophotometric measurements. Neither ethanol nor water is listed as PBT or hazardous. The pH is not corrosive and the produced waste is <50 g per sample. Therefore, the proposed method passes the four criteria, and the four quadrants of the greenness profile are green.

#### 3.4.2. Analytical Eco-Scale

In this method a more quantitative assessment is presented, where a numerical score is calculated to assess the method greenness, and high Eco-Scale score (i.e., > 75) denotes the method greenness. Whenever the method uses hazardous chemicals, generates waste, has high energy consumption, or has exposure risk, penalty points are deducted from the total 100 points [47]. The high Eco-Scale score of the proposed methods (95) indicates the excellent greenness of the developed method (Table 10). Thus, they can be employed for the routine analysis of the studied drugs in pharmaceutical formulations and urine samples with very few harmful effects on the environment.

## 4. Conclusions

Rapid, accurate, and cost-effective green nonconventional univariate and multivariate chemometrics-assisted spectrophotometric methods were developed for the determination of AT, PR, HZ, and LV in different pharmaceutical dosage forms. In addition, the proposed methods were successfully applied for the determination of the four analytes in spiked and real human urine samples and both methods provided comparable results. The assessment of the greenness profile of the two methods showed they represent an excellent green analysis of the studied drugs with very low harmful effects on the environment. Moreover, the proposed methods do not need any sample preparation, sophisticated HPLC instrumentation, or expensive solvents. Furthermore, the EDR method has the advantage of being simple and does not need any sophisticated mathematical algorithms needed for the MCR method. However, the main challenge in developing the EDR method is the selection of the optimum divisor to get selective and reproducible results. MCR-ALS method has the advantage over other first-order multivariate calibration methods of being able to produce good prediction even in the presence of inferences that are not represented in the calibration phase. The proposed methods can be used as a valid eco-friendly and simple cost-effective alternative to the commonly used chromatographic methods for the routine analysis of the studied drugs in dosage forms and human urine.

## Figures and Tables

**Table 1:** Characteristic validation parameters of the proposed EDR method for the determination of AT, PR, HZ, and LV.

Parameters	AT	PR	HZ	LV
Calibration range (*μ*g mL^−1^)	5–40	1–25	1–15	1–15
Regression equation:				
Slope (*b*)	0.0053	0.1830	0.1211	0.0998
Intercept (*a*)	−1.43 × 10^−4^	−3.23 × 10^−4^	−6.10 × 10^−3^	−1.37 × 10^−3^
Correlation coefficient (*r*^2^)	0.9995	0.9998	0.9994	0.9996
Standard deviation of slope (*S*_*b*_)	5.19 × 10^−5^	1.24 × 10^−3^	1.32 × 10^−3^	8.50 × 10^−4^
Standard deviation of intercept (*S*_*a*_)	1.22 × 10^−3^	1.75 × 10^−2^	1.18 × 10^−2^	7.66 × 10^−3^
LOD^a^ (*μ*g mL^−1^)	0.76	0.31	0.32	0.25
LOQ^b^ (*μ*g mL^−1^)	2.31	0.95	0.98	0.77
Accuracy (mean ± SD^c^)	99.82 ± 0.88	99.93 ± 0.85	99.98 ± 0.69	100.26 ± 0.77
Precision:				
Intraday (% RSD^d^)	0.70	0.66	0.93	0.98
Interday (% RSD^e^)	1.26	0.88	0.90	0.94

^a^Limit of detection. ^b^Limit of quantification. ^c^Standard deviation of three different concentrations each analyzed in triplicate. ^d^Relative standard deviation of three different concentrations analyzed in triplicate within the same day. ^e^Relative standard deviation of three different concentrations analyzed in triplicate on three consecutive days.

**Table 2:** Determination of AT, PR, HZ, and LV in synthetic mixtures using the proposed EDR method.

Mix. No.	Conc. (*μ*g mL^−1^)				% Recovery			
	AT	PR	HZ	LV	AT	PR	HZ	LV
1	15	10	3	6	98.15	99.70	100.70	101.5
2	30	2	5	5	97.85	99.15	99.70	99.40
3	25	6	8	4	100.20	101.20	98.45	100.00
4	25	5	7	4	99.56	100.91	101.62	100.25
5	10	1	4	1	102.11	98.70	101.90	99.10
6	15	4	5	3	98.40	100.00	99.80	99.80
Mean					99.38	99.94	100.36	100.01
± SD					1.61	0.97	1.30	0.84

**Table 3:** Statistical comparison of the proposed EDR method with published methods for the determination of AT, PR, HZ, and LV.

	AT		PR		HZ		LV	
	EDR	Published method [3]	EDR	Published method [16]	EDR	Published method [28]	EDR	Published method [34]
Mean	99.77	100.05	101.04	100.11	101.07	100.30	99.69	100.15
± SD	1.10	1.06	1.16	1.05	0.71	0.96	1.22	1.12
n	3	3	3	3	3	3	3	3
*t* ^a^	0.23		1.32		1.08		1.63	
*F* ^a^	1.08		1.21		1.86		1.19	

^a^Critical values of *t* and *F* are 4.30 and 19.00, respectively, at (*p*=0.05).

**Table 4:** The concentration matrix used for preparation of the calibration and validation sets for the MCR-ALS method.

Mix. no.	AT	PR	HZ	LV
1	15	5.5	5.5	4
2	15	1	1	7
3^*∗*^	5	1	10	2.5
4^*∗*^	5	10	3.5	7
5	25	3.5	10	4
6	10	10	5.5	2.5
7	25	5.5	3.5	2.5
8	15	3.5	3.5	5.5
9^*∗*^	10	3.5	8	7
10	10	8	10	5.5
11	20	10	8	4
12	25	8	5.5	7
13	20	5.5	10	7
14	15	10	10	1
15^*∗*^	25	10	1	5.5
16	25	1	8	1
17	5	8	1	4
18^*∗*^	20	1	5.5	5.5
19	5	5.5	8	5.5
20^*∗*^	15	8	8	2.5
21	20	8	3.5	1
22	20	3.5	1	2.5
23^*∗*^	10	1	3.5	4
24	5	3.5	5.5	1
25	10	5.5	1	1

^*∗*^Mixtures used as external validation set.

**Table 5:** Figures of merit of the MCR-ALS model for the calibration set of AT, PR, HZ, and LV.

Parameters	AT	PR	HZ	LV
Calibration range (*μ*g mL^−1^)	5–25	1–10	1–10	1–7
Slope	0.9998	1.0000	1.0001	1.0014
Standard error of slope	3.28 × 10^−3^	3.00 × 10^−3^	2.80 × 10^−3^	1.88 × 10^−3^
Intercept	3.27 × 10^−3^	4.96 × 10^−4^	−7.84 × 10^−5^	−3.29 × 10^−3^
Standard error of intercept	5.67 × 10^−2^	1.95 × 10^−2^	1.81 × 10^−2^	8.00 × 10^−3^
RMSEC	9.08 × 10^−2^	3.34 × 10^−2^	3.67 × 10^−2^	1.66 × 10^−2^
SEP	8.83 × 10^−2^	3.25 × 10^−2^	3.56 × 10^−2^	1.62 × 10^−2^
Bias	−7.15 × 10^−4^	−3.90 × 10^−4^	−3.00 × 10^−4^	−2.01 × 10^−3^
RE (%)	0.526	0.515	0.567	0.391
Determination coefficient (*r*^2^)	0.9998	0.9999	0.9999	0.9999

**Table 6:** Figures of merit of the MCR-ALS model for the validation set of AT, PR, HZ, and LV.

Parameters	AT	NP	HZ	LV
Accuracy (mean ± SD)^a^	99.71 ± 1.16	99.71 ± 1.22	99.94 ± 0.85	100.01 ± 0.79
Precision:				
Intraday (% RSD)^b^	1.57	1.32	1.35	0.86
Interday (% RSD)^c^	1.70	1.24	1.65	0.72
RMSEP	0.2033	0.0307	0.0461	0.0366
SEP	0.1882	0.0284	0.0426	0.0339
Bias	6.63 × 10^−2^	−8.34 × 10^−3^	−1.08 × 10^−3^	2.60 × 10^−3^
RE (%)	1.39	0.49	0.72	0.71
*r* ^2^	0.9994	0.9999	0.9999	0.9996

^a^Mean and standard deviation of 7 determinations. ^b^Relative standard deviation of three different concentrations analyzed in triplicate within the same day. ^c^Relative standard deviation of three different concentrations analyzed in triplicate on three consecutive days.

**Table 7:** Determination results of AT, PR, HZ, and LV in dosage forms by the proposed and reference methods.

Parameter		Conc. Claimed (*μ*g mL^−1^)	EDR		MCR-ALS		Reference method^*∗*^	
			Conc. Found (*μ*g mL^−1^)	% Recovery	Conc. Found (*μ*g mL^−1^)	% Recovery	Conc. Found (*μ*g mL^−1^)	% Recovery
AT	Tenormin® tablets	10	10.05	100.50	9.96	99.60	10.10	101.00
		15	14.85	99.00	15.05	100.33	15.08	100.53
		20	19.61	98.05	19.75	98.75	19.9	99.50
	Mean			99.18		99.56		100.34
	± SD^a^			1.24		0.79		0.77
	*t* ^b^			3.50		2.26		
	*F* ^b^			2.59		1.07		

PR	Panadrex® tablets	3	2.96	98.67	3.02	100.67	2.98	99.33
		5	5.05	101.00	4.93	98.60	5.03	100.60
		10	10.04	100.40	10.1	101.00	9.8	98.00
	Mean			100.02		100.09		99.31
	± SD^a^			1.21		1.30		1.30
	*t* ^b^			0.79		0.53		
	*F* ^b^			1.15		1.00		

HZ	HCT georetic 25® tablets	3	3.03	101.00	3.01	100.33	3.04	101.33
		5	4.92	98.40	4.96	99.20	4.98	99.60
		10	9.88	98.80	10.11	101.10	9.92	99.20
	Mean			99.70		99.77		100.47
	± SD^a^			1.84		0.80		1.23
	*t* ^b^			2.31		0.19		
	*F* ^b^			0.66		1.41		

LV	Tavanic® tablets	3	2.98	99.33	3.01	100.33	2.98	99.33
		4	4.03	100.75	4.04	101.00	4.02	100.50
		5	5.03	100.60	5.01	100.20	5.03	100.60
	Mean			100.23		100.51		100.14
	± SD^a^			0.78		0.43		0.70
	*t* ^b^			1.00		0.89		
	*F* ^b^			1.22		2.70		

^a^Standard deviation of the mean of percentage recovery from the label claim amount. ^b^Theoretical values for *t* and *F* are 4.30 and 19.00 at (*p*=0.05), respectively. ^*∗*^Reference methods for AT [3], PR [16], HZ [28] and LV [34].

**Table 8:** Determination results of AT, PR, HZ, and LV in spiked human urine samples using the proposed methods.

Sample no.	Conc. (*μ*g mL^−1^)				EDR				MCR-ALS			
	AT	PR	HZ	LV	AT	PR	HZ	LV	AT	PR	HZ	LV
1	25	3	3.5	4	104.10	102.90	103.15	102.9	99.88	99.20	101.83	102.64
2	10	5	4	1	97.81	101.30	104.7	105.35	101.41	97.94	100.86	102.12
3	15	7	2	3	102.15	103.70	103.71	98.11	102.77	96.86	98.69	101.04
Mean					101.35	102.63	103.85	102.12	101.35	98.00	100.46	101.93
± SD					3.22	1.22	0.78	3.68	1.45	1.17	1.61	0.82

**Table 9:** Determination of urinary excretion of PR and LV after administration of a single 500 mg oral dose of PR or LV by healthy volunteers using EDR and MCR-ALS methods.

Compound	Parameter	EDR	MCR-ALS	Reference method^*∗*^
PR	Concentration (*μ*g mL^−1^)	454.5	459.7	457.4
	Excretion (0–12 h) (mg)	445.4	450.5	448.2
	% Excretion (0–12 h)	89.1	90.1	89.6

LV	Concentration (*μ*g mL^−1^)	315.3	324.9	318.5
	Excretion (0–12 h) (mg)	302.7	311.9	305.8
	% Excretion (0–12 h)	60.5	62.4	61.2

^*∗*^Reference methods: PR [16] and LV [34].

**Table 10:** Analytical Eco-Scale score for the proposed methods.

Method items	Value	Penalty points (PPs)
Reagents		
(Water) Reagent amount	<10 mL	1
Reagent hazardous	None	0
(Ethanol) Reagent amount	<10 mL	1
Reagent hazardous		2
**Total PPs (PPs of reagent amount** **×** **PPs reagent hazard)**		**2**
**Instruments**		
Energy	<0.1 kWh per sample	0
Occupational hazards	Emission of vapors and gases to the air	0
Waste	1–10 mL	3
**Total PPs (sum of instrument PPs)**	**3**	

**Total PPs (PPs reagent** **+** **PPs instrument)**		5
**Analytical Eco-Scale total score**	100 − 5 = 95	**95**

## Data Availability

The data used to support the findings of this study are available from the corresponding author upon request.
